# Genome-wide identification of *Solanum tuberosum* L. AAAP gene family and its response to abiotic stress

**DOI:** 10.1371/journal.pone.0333560

**Published:** 2025-10-09

**Authors:** Zhichang Gao, Xinrui Zhao, Yanqun Wang, Xiaotian Chen, Lei Wang, Xiaojing Liu, Jianghui Cui

**Affiliations:** 1 College of Agronomy, Hebei Agricultural University, Baoding, China; 2 Zhuolu County Agricultural and Rural Bureau Agricultural (Agricultural Mechanization) Technology Promotion Station, Zhangjiakou, China; 3 Agriculture and Rural Bureau of Weichang Manchu and Mongolian Autonomous County, Chengde, China; 4 Weichang Manchu and Mongolian Autonomous County Potato Research Institute, Chengde, China; University of Delhi, INDIA

## Abstract

The amino acid/auxin permease (AAAP) protein is an amino acid transporter involved in many biological processes in plants, especially in plant responses to abiotic stress. This study systematically identified potato StAAAP gene family, revealed its characteristics, and analyzed its functions in potato resistance to abiotic stress. Furthermore, its gene structure, chromosome distribution, cis-acting elements, conserved protein moieties, and collinearity between species were analyzed. The expression pattern of StAAAP in potato plants under abiotic stress was analyzed using RNA-seq data downloaded from the National Center for Biotechnology Information (NCBI), and the gene expression pattern was verified using qRT-PCR. A total of 56 members of the StAAAP gene family were identified in potato and were distributed across 12 chromosomes. Based on their phylogenetic characteristics, they were divided into eight subfamilies: ATLa, ATLb, AAP, ANT, AUX, GAT, LHT, and ProT. The gene structure and conserved motifs of members of the same subfamily are essentially the same, and the AAAP gene family members are mostly distributed in the plasma membrane. Potato StAAAP family members contain a large number of cis-acting elements related to the stress response. Collinearity analysis revealed a large number of homologous gene pairs in the potato, tomato, pepper, and tobacco AAAP families. Expression analysis revealed that StAAAP family members were highly expressed under drought and salt stress conditions, and the expression of the same gene was different in different family members. The genes StAAAP4, StAAAP24, StAAAP29, StAAAP40, and StAAAP46 may play key roles in the abiotic stress response of potatoes. StAAAP genes play an important role in the growth, development, and abiotic stress responses of potato plants.

## 1. Introduction

Studies have shown that the amino acid/auxin permease (AAAP) protein is an amino acid transporter used for long-distance transport [1,2]. Amino acids, mainly in the form of organic nitrogen, participate in protein synthesis, metabolism, hormone regulation, and other life processes in plants [3–5]. This is the communication link between the cells and tissues in multicellular organisms [6]. Amino acids must be transported among the different organs of the plant to function; however, amino acids cannot autonomously cross the structure of the cell membrane, and this process requires the help of amino acid transporters [7]. The realization of these functions requires amino acid transport systems. The largest family of amino acid transporters includes the amino acid/auxin permease (AAAP) family [2], which can transport auxin (indole-3-acetic acid), C-aminobutyric acid, mono-L-amino acid, and multiple amino acids and participate in the long-distance amino acid transport process of plants, thereby playing a crucial role in the growth and development of plants. Based on the similarity between sequences and the characteristics of the conserved domains, AAAP can be divided into eight branches. These include amino acid permease (AAP), lysine and histidine transporter (LHT), gamma-aminobutyric acid transporter (GAT), proline transporter (ProT), auxin transporter (ANT), aromatic and neutral amino acid transporter (ANT), amino acid transporter-like a (ATLa),and amino acid transporter-like b (ATLb) [7–9]. Sequence similarity between different subfamilies was low, but all AAAP proteins shared the same conserved domain, the Aa_trans domain (PF01490) [10].

AAAP is an important amino acid transporter whose gene family members are widely distributed in plants, animals, fungi, and eukaryotes [6]. At present, we have identified some functions of AAAP proteins in plants, such as in Arabidopsis [11], poplar [12], rice [13], and tobacco [14]. In Arabidopsis, AtAAP1 mediates the absorption of amino acids by embryo or root cells [1,4,15]. In tobacco, NtAAP2−2 is involved in the transport of asparagine, aspartate, glutamine, and glutamate [14]. In poplar, PtAAP11 is involved in xylem proline transport [16]. In rice, OsAAP6 can promote the increase of grain protein content (GPC) and absorption of amino acids by root system, as well as affect the distribution of amino acids [17]. OsAAP4 can also improve tillering and grain yield by increasing the neutral amino acid concentrations of valine (Val), proline (Pro), threonine (Thr), and leucine (Leu) in rice [18].

Potato (*Solanum tuberosum* L.) is a tuber belonging to the Solanaceae family and an important food source in many regions. We know from previous studies that potatoes contain a total of 72 amino acid transporters (AATs) [19]. These play an important role in regulating amino acid absorption and protein content during seed and tuber development [19]. StAAP1 is expressed during the conversion of source to sink, and in transgenic plants with reduced StAAP1 expression, free amino acid content is almost halved [20]. Although potato AATs have been studied previously, the present study analyzed the potato AAAP gene in different abiotic stress environments. The AAAP gene family members in potato were identified using bioinformatics, and the chromosome distribution, gene structure, evolutionary characteristics, and expression patterns of the AAAP genes were systematically analyzed. In addition, we retrieved publicly available high-throughput transcription data, screened candidate StAAAP genes involved in amino acid content transport and verified them using quantitative real-time RT-PCR (qRT-PCR), providing a theoretical basis for exploring the role of potato AAAP genes in abiotic stress.

## 2. Materials and methods

### 2.1. Materials

This study selected the ‘Jinong Potato 8511’ independently cultivated by Hebei Agricultural University as the experimental material. It is a homologous tetraploid variety. The growth period of this variety is approximately 105 days. The tubers have yellow skin and flesh. It has moderate drought resistance and salt tolerance. By 2023, it was registered as a non-major crop variety by the Ministry of Agriculture and Rural Affairs. The experiment was conducted in the laboratory of the College of Agriculture at Hebei Agricultural University.

### 2.2. Methods

#### 2.2.1. Identification of AAAP family members of potato and analysis of their physicochemical properties.

Genomic data of potato (*Solanum tuberosum* L.) was obtained from the Ensembl database (http://plants.ensembl.org/index.html), and the AAAP Hidden Markov Model (HMM) (PF01490) was retrieved from the Pfam database (http://pfam.xfam.org/).The AAAP structure domain was screened using the HMMER 3.1 software (http://hmmer.org/download.html), and an E-value < 1e^-5^ was selected. The candidate AAAP family members were identified using the pfam (http://pfam.xfam.org/), NCBI CDD (https://www.ncbi.nlm.nih.gov/cdd/), and SMART (http://smart.embl.de/) databases; three other major databases further confirmed the potato AAAP domain, and only members containing the AAAP domain were identified as AAAP family members.

The number of amino acids, molecular weight, isoelectric point, and other physical and chemical properties of the potato AAAP family proteins were analyzed using ExPASy ProtParam (http://web.(expasy.org/protparam/) [21].

#### 2.2.2. Analysis of AAAP gene family evolution, conserved motifs, and gene structure.

ClustalW in MEGA7.0 [22] was used to compare the all-length AAAP egg white sequences in the species based on the neighbor-joining method, and the bootstrap value was set to 1,000. Using the remaining default parameters, a phylogenetic tree of the AAAP family was plotted.

CFVisual software was used to predict the domain according to the potato AAAP family GFF file and the CDD website, and the resulting file included a gene structure map as well as a domain distribution map. The conserved domain and gene structure of potato AAAP family members were predicted using MEME (http://meme-suite.org) and GSDS2.0 (http://gsds.gao-lab.org/).

#### 2.2.3. Analysis of the position of genes on chromosomes and collinearity.

Information on potato AAAP family genes on chromosomes was obtained from the potato genome sequence DNA annotation file, and the genome distribution map was drawn using the TbtoolsII software.

The internal collinearity of potatoes was analyzed using the Python version of MCScanX software, and the results were visualized using Circos-v0.69 software and the drawing program.

#### 2.2.4. Cis-acting element analysis.

The sequence length of 2000 bp upstream of potato AAAP family gene was extracted as the promoter sequence. The PlantCARE (http://bioinformatics.psb.ugent.be/webtools/plantcare/html/) database was used for cis element analysis of the potato AAAP gene family.

#### 2.2.5. Transcriptome sequencing.

Using RNA-seq data downloaded from the NCBI database, the expression patterns of AAAP genes in potato were analyzed under different abiotic stresses: salt stress, 200 mmol/L NaCl treatment for 12 and 24 h (entry number: PRJNA882516); Simulated drought stress, 150 mmol/L mannitol treatment for 12 and 24 h (entry number: PRJNA646050). A gene expression calorimetric map was drawn using the Tbtools II software.

#### 2.2.6. qRT-PCR verification.

Potato seedlings of the selected materials were cultured in vitro. When the seedlings were grown for 21 d, simulated drought stress was carried out under PEG-6000 treatment at a concentration of 25 mmol/L, whereas salt stress was carried out under NaCl treatment at a concentration of 70 mmol/L. Whole plant samples were collected at 0, 12, and 24 h after stress. Before RNA extraction, the samples were frozen in liquid nitrogen and then stored at −80°C.

Total RNA was extracted using a Promeg RNA extraction kit (LS1040), and the integrity and concentration of the RNA were determined using agarose gel electrophoresis and a Nanodrop ND-2000 spectrophotometer (Nanodrop Technologies, USA). Subsequently, first-strand cDNA was synthesized by reverse transcription using the KW RT gDNA kit (CW2020M). RT-qPCR was performed on a CFX96 (Bio-Rad, USA) using the US EVERBRIGHT (AugeGreen qPCR Master Mix S2008L) kit with three biological and three technical replicates. Reaction conditions were based on the AugeGreen qPCR Master Mix product specifications. *StEF-1α*was used as the internal reference gene [23]. The relative expression of StAAAP genes was calculated using 2^-ΔΔct^ [24]. The primers were synthesized by Shenggong Bioengineering Co., Ltd. (Shanghai, China).

## 3. Results

### 3.1. StAAAP gene identification

In this study, 56 AAAP genes were identified in the whole potato genome and named StAAAP 1–56 according to their chromosomal locations. The results are shown in Table 1.To characterize the StAAAP genes, we analyzed their open reading frame length, protein sequence, theoretical isoelectric point (pI), protein molecular weight (MW), and subcellular and chromosomal locations (Table 1). The StAAAP genes range from 249 bp (StAAAP1) to 1827 bp (StAAAP55), with an average length of 1328 bp. Among the 56 StAAAP genes, the longest potato U-box protein is StAAAP55, which contains 608 amino acids, and the shortest is StAAAP1, which contains 82 amino acids. The molecular weights of the proteins range from 9321 Da to 67956.4 Da, with an average of 48,724.1 Da. The pI ranges from 4.63 (StAAAP17) to 10.24 (StAAAP1). WoLF PSORT predicted the subcellular location of StAAAP. We found most AAAP proteins (56) in the cytoplasm (6), plasma membrane (41), and vacuole (4). In addition, two were found in the nucleus, and three in the chloroplast.

**Table 1:** Analysis of potato AAAP gene family members and physicochemical properties.

Gene Name	Gene ID	Chromosome localization			Protein length (aa)	MW (Da)	pI	CDS length	Subcellular localization
		Chr	Start	End					
StAAAP1	PGSC0003DMG400025834	Chr01	83425096	83425713	82	9321	10.24	249	Nucleus
StAAAP2	PGSC0003DMG400001589	Chr01	87286988	87291068	494	55706.5	8.63	1485	Cytoplasm
StAAAP3	PGSC0003DMG400018651	Chr01	87962706	87965704	563	61354.5	9.5	1692	Plasma membrane
StAAAP4	PGSC0003DMG400017473	Chr02	19129779	19132621	421	46260.6	8.67	1266	Chloroplast
StAAAP5	PGSC0003DMG400026549	Chr02	24907881	24913763	463	50377.8	8.54	1392	Plasma membrane
StAAAP6	PGSC0003DMG400035823	Chr02	38385229	38386506	425	46927.3	7.38	1278	Plasma membrane
StAAAP7	PGSC0003DMG400043788	Chr02	38387386	38388654	422	46449.3	4.85	1269	Cytoplasm
StAAAP8	PGSC0003DMG400001474	Chr02	45187534	45192800	482	52638.4	8.77	1449	Plasma membrane
StAAAP9	PGSC0003DMG400020266	Chr02	47763577	47766815	449	50363	8.77	1350	Plasma membrane
StAAAP10	PGSC0003DMG400020265	Chr02	47774685	47776859	445	49900.4	8.95	1338	Plasma membrane
StAAAP11	PGSC0003DMG400018670	Chr03	3106659	3108625	427	47068.3	7.9	1284	Plasma membrane
StAAAP12	PGSC0003DMG400013962	Chr03	16125457	16131700	444	48650.1	4.91	1335	Plasma membrane
StAAAP13	PGSC0003DMG400031456	Chr03	17197666	17201379	449	49308.1	9.58	1350	Plasma membrane
StAAAP14	PGSC0003DMG400029866	Chr03	26824152	26830361	420	46221.4	8.23	1263	Plasma membrane
StAAAP15	PGSC0003DMG400029867	Chr03	27067967	27069204	336	36721.2	8.42	1011	Vacuole
StAAAP16	PGSC0003DMG400003397	Chr03	29797320	29799530	426	47061.2	7.37	1281	Plasma membrane
StAAAP17	PGSC0003DMG400031347	Chr03	31760106	31768665	523	57260.9	4.63	1572	Plasma membrane
StAAAP18	PGSC0003DMG400013982	Chr03	34431996	34436659	441	48627.8	9.51	1326	Plasma membrane
StAAAP19	PGSC0003DMG400013981	Chr03	34488013	34494453	439	48669.6	9.03	1320	Plasma membrane
StAAAP20	PGSC0003DMG400015198	Chr03	50596476	50600303	416	45994.2	9.51	1251	Plasma membrane
StAAAP21	PGSC0003DMG400014203	Chr03	57084598	57086764	472	51262.5	5.25	1419	Plasma membrane
StAAAP22	PGSC0003DMG400006341	Chr04	7319379	7322409	408	45303.7	9.28	1227	Cytoplasm
StAAAP23	PGSC0003DMG400004961	Chr04	66074335	66079024	481	52741.8	8.66	1446	Plasma membrane
StAAAP24	PGSC0003DMG401004944	Chr04	66527155	66537044	451	48987.7	8.14	1356	Plasma membrane
StAAAP25	PGSC0003DMG402004944	Chr04	66532212	66536643	398	43341.5	7.53	1197	Plasma membrane
StAAAP26	PGSC0003DMG400011690	Chr04	68864668	68870114	525	57858.1	9.59	1578	Plasma membrane
StAAAP27	PGSC0003DMG402009896	Chr04	71461110	71463731	161	18041.4	9.52	486	Plasma membrane
StAAAP28	PGSC0003DMG400009969	Chr04	71461110	71466108	509	56070.7	8.46	1530	Nucleus
StAAAP29	PGSC0003DMG400000818	Chr05	2125547	2129338	438	48991.3	8.88	1317	Plasma membrane
StAAAP30	PGSC0003DMG400017438	Chr05	12017643	12020428	447	50244.1	9.72	1344	Plasma membrane
StAAAP31	PGSC0003DMG400003352	Chr05	41731287	41732681	464	50802.7	8.15	1395	Plasma membrane
StAAAP32	PGSC0003DMG400016801	Chr05	46491441	46492847	468	51017.2	8.37	1407	Plasma membrane
StAAAP33	PGSC0003DMG400011639	Chr05	47556832	47559928	438	48120.1	8.96	1317	Cytoplasm
StAAAP34	PGSC0003DMG400027187	Chr05	48090781	48095798	438	48248.2	9.73	1317	Plasma membrane
StAAAP35	PGSC0003DMG400027188	Chr05	48097039	48104535	452	49362.3	9.58	1359	Plasma membrane
StAAAP36	PGSC0003DMG400029154	Chr06	30893598	30898031	440	48198.4	6.67	1323	Plasma membrane
StAAAP37	PGSC0003DMG400029153	Chr06	30910842	30913080	440	48120	9.07	1323	Vacuole
StAAAP38	PGSC0003DMG400011771	Chr06	42779953	42785063	473	51861.1	8.22	1422	Plasma membrane
StAAAP39	PGSC0003DMG400007675	Chr06	44978943	44979587	150	16709	9.92	453	Vacuole
StAAAP40	PGSC0003DMG400022256	Chr07	55966125	55970593	456	50599.8	8.29	1371	Plasma membrane
StAAAP41	PGSC0003DMG400022255	Chr07	55971749	55976656	472	51978.5	8.84	1419	Plasma membrane
StAAAP42	PGSC0003DMG400012297	Chr08	55721575	55725165	295	32249.9	9.52	888	Chloroplast
StAAAP43	PGSC0003DMG400008504	Chr09	210407	214610	481	54327.9	8.25	1446	Plasma membrane
StAAAP44	PGSC0003DMG400021623	Chr09	61205870	61207293	369	40354.9	10.02	1110	Chloroplast
StAAAP45	PGSC0003DMG400012842	Chr10	13369133	13370690	432	47131.5	5.68	1299	Cytoplasm
StAAAP46	PGSC0003DMG400023205	Chr10	46744707	46751963	488	54970.8	8.75	1467	Plasma membrane
StAAAP47	PGSC0003DMG400010866	Chr10	47784911	47788720	496	55143.3	8.15	1491	Cytoplasm
StAAAP48	PGSC0003DMG400019182	Chr10	50480301	50485816	485	54606.2	7.97	1458	Plasma membrane
StAAAP49	PGSC0003DMG400011068	Chr10	54327353	54331948	529	57499.1	4.83	1590	Plasma membrane
StAAAP50	PGSC0003DMG400008360	Chr10	59202658	59204878	518	55014.2	9.69	1557	Vacuole
StAAAP51	PGSC0003DMG400007424	Chr11	3581626	3586016	602	66619.1	7.54	1809	Plasma membrane
StAAAP52	PGSC0003DMG400006021	Chr11	8463730	8466858	479	52924.3	8.76	1440	Plasma membrane
StAAAP53	PGSC0003DMG400006550	Chr11	10041987	10046875	468	53198.7	8.83	1407	Plasma membrane
StAAAP54	PGSC0003DMG400000468	Chr11	39686792	39693985	453	49711.9	9.1	1362	Plasma membrane
StAAAP55	PGSC0003DMG400017687	Chr12	13896229	13902666	608	67956.4	7.89	1827	Plasma membrane
StAAAP56	PGSC0003DMG400011538	Chr12	56031020	56036743	488	54099.6	8.85	1467	Plasma membrane

The family members of the potato AAAP genes were analyzed, and a total of 56 family members were identified. On the left side, 56 family members are numbered StAAAP1–56. Each column shows the chromosomal localization (including initial and terminal lengths), protein length (aa), molecular weight (Da), pI, CDS length, and subcellular localization of these family members.

### 3.2. Phylogenetic analysis and classification of StAAAP gene family members

**Fig 1:**
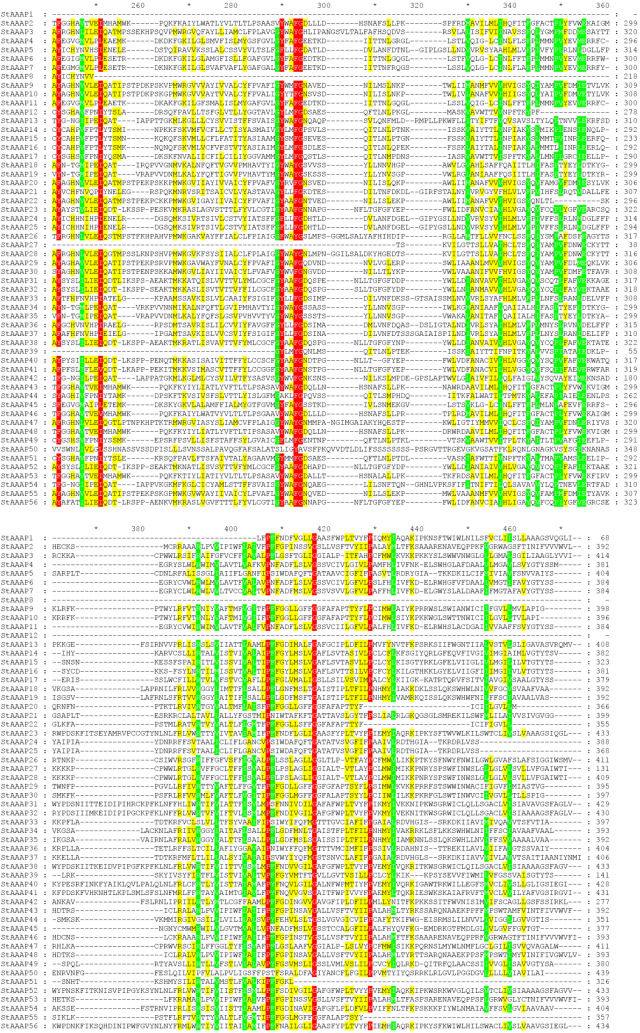
Multiple sequence alignment of the StAAAPs. Multiple alignments were performed based on the protein domain sequences of the 56 StAAAP family members. Through analysis of similarities and differences in amino acid sequences, the conserved functional regions of the families were explored. Red, green, and yellow marks indicate that the conservation of amino acid residues was greater than 90%, 70%, and 50%, respectively. Highly conserved column (marked in red): This region is irreplaceable within the StAAAP family and may be a key functional area, such as substrate-binding sites and catalytic domains.

A multi-sequence alignment analysis was conducted on the domains of 56 StAAAP family members,with the results shown in Fig 1. Colors are used to visually distinguish differences in the conservation of amino acid sequences. The conservation of the red-marked region exceeded 90% and the amino acids in multiple sequences were consistent, suggesting a highly conserved functional core domain, which might be a substrate-binding or catalytic active site. The conservation of the green region exceeded 70% and the sequence consistency was relatively high, thus participating in maintaining the basic structure of the protein. The conservation rate in the yellow area exceeded 50% and amino acid variation was relatively obvious. Regions with consistent colors in the same column show the common functional modules of the family, whereas sites with mixed colors reflect large sequence variations, which may be related to protein functional differentiation or species-specific adaptation, providing key clues for analyzing the evolutionary relationships and functional diversity of the StAAAP family.

**Fig 2:**
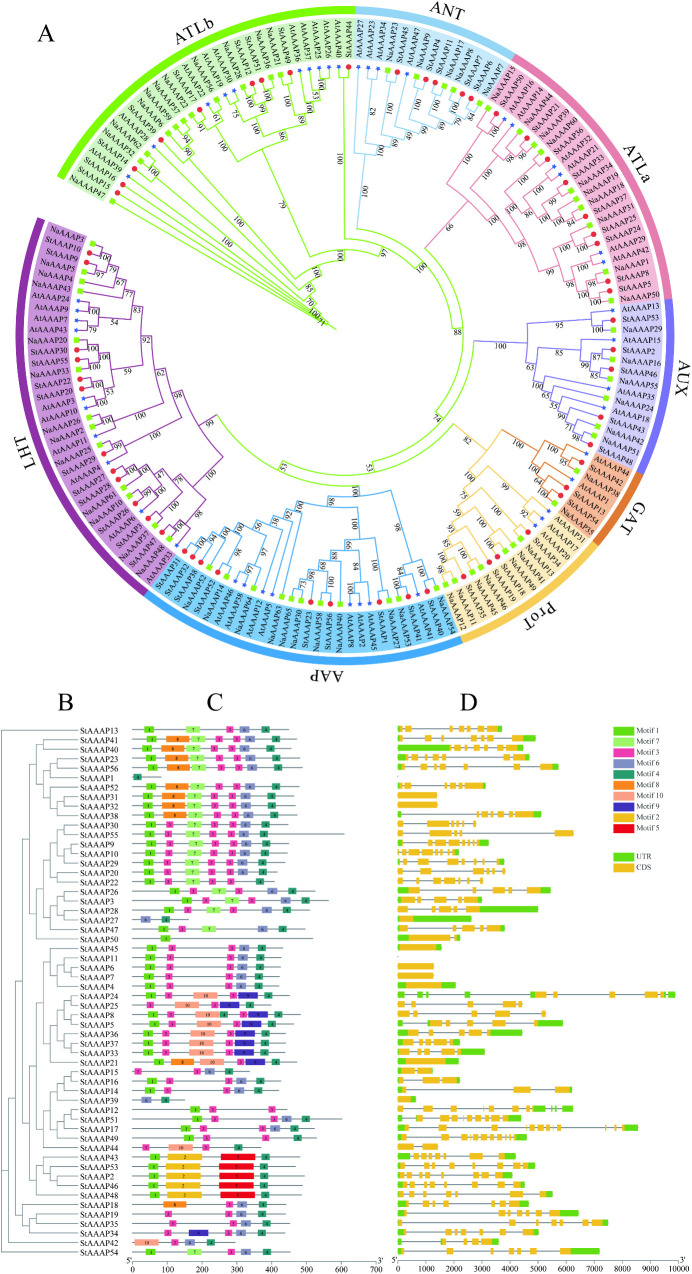
Phylogenetic, gene structure, and conserved motif analyses of the AAAP gene family. A: Phylogenetic trees of the AAAP families in *S. tuberosum*, *A. thaliana,* and *N. attenuata*. B Phylogenetic tree of the StAAAP family. C: Analysis of the conserved motif of StAAAP D: Analysis of StAAAP gene structure. Phylogenetic trees of potato, *A. thaliana,* and tobacco were constructed, and the AAAP gene family was divided into eight subfamilies. The motifs of the StAAAP genes were evaluated, and 10 motifs were identified among the 56 StAAAP genes. The intron-exon structures of the StAAAP members in potatoes were further analyzed.

To further understand the homology between potato and other plant AAAP gene families, we constructed rootless phylogenetic trees of full-length AAAP in potato (56 genes), *Arabidopsis thaliana* (47 genes), and tobacco (65 genes) as shown in Fig 2A–2B. The eight subfamilies(ATLa, atb, AAP, ANT, AUX, GAT, lt and ProT) of the StAAAP, AtAAAP and NaAAAP genes are shown in Table 2. LHT had the largest number of AAAP genes with 35 genes, followed by ATLb and AAP with 30 and 28 genes, respectively. GAT had the smallest number of AAAP genes with seven genes (Table 2). The number of genes in potato and tobacco was close in multiple subfamilies, and the genetic relationship of the AAAP gene family was close. These results further support the accuracy of the phylogenetic analysis of the StAAAP gene family in this study.

**Table 2:** Number of family members of each species in the AAAP family.

Group	Species			Total number
	Potato	*Arabidopsis thaliana*	Tobacco	
ATLa	9	6	10	25
ATLb	9	10	11	30
AAP	9	8	11	28
ANT	5	4	5	14
AUX	5	4	6	15
GAT	3	2	2	7
LHT	12	10	13	35
ProT	4	3	7	14
Total number	56	47	65	168

The AAAP gene family comprises eight subfamilies. The distribution and quantity of AAAP genes in eight potato subfamilies, *A. thaliana*, and tobacco subfamilies were analyzed. The results showed that the number of genes in potato and tobacco was close in multiple subfamilies.

To further study the relationship of the StAAAP gene family in the phylogenetic tree, the StAAAP gene motif was evaluated using MEME and the results are shown in Fig 2C. Ten motifs were identified in 56 StAAAP genes. Motifs 1, 3, and 4 were found in all StAAAP members, indicating that they are highly conserved in StAAAP protein. Genes in the same subfamily in the phylogenetic tree exhibited similar motifs, indicating that they may have similar functions. For example, almost all AAAP family members in the AAP subfamily have four motifs (1, 3, 4, and 6). Similarly, all AAAP family members in the GAT subfamily also have four motifs (1, 2, 4, and 5).

To better understand the composition and function of StAAAP genes, we performed gene structure analysis of its conserved sequences and intron positions (Fig 2D). The results showed that potato AAAP genes undergo a complex RNA splicing process, with exon numbers ranging from 2 to 13. Genes in the same subfamily have similar exon/intron structures, whereas there are differences in the gene structure among different subfamilies. All five genes in the ANT subfamily contain one exon, which is the smallest of the eight gene groups. The AUX subfamily contained most of the exons, with an average of eight exons per gene. Gene structure and motif analyses were consistent with those of the StAAAP phylogenetic tree, providing strong evidence for the accuracy of the phylogenetic tree classification.

### 3.3. Chromosomal localization and homologous gene analysis of StAAAP genes

**Fig 3:**
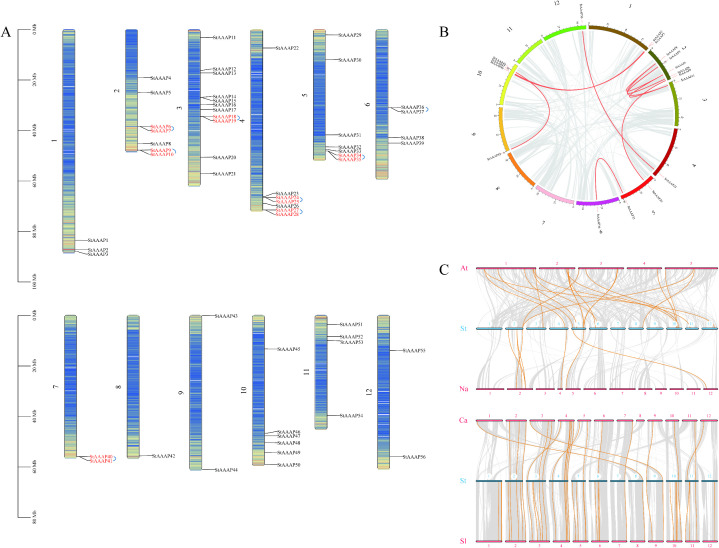
Gene duplication events in the AAAP family of *S. tuberosum.* A: Chromosomal localization map of StAAAP genes. B: Collinearity analysis of StAAAP genes. C: Collinearity analysis of AAAP genes in multiple species (At: *A. thaliana*; St: *S. tuberosum*; Na: *N. attenuata*; Ca: *C. annuum*; and Sl: *S. lycopersicum*).. Panel A shows that 56 StAAAP genes were non-uniformly and non-randomly located on 12 chromosomes, and eight pairs of tandem repeat genes were identified. Panel B shows that there are a total of 10 pairs of collinear genes for the StAAAP genes in potato. Panel C shows the interspecific collinear maps of the potato AAAP family genes with *A. thaliana*, pepper, tobacco, and tomato, and the interspecific evolutionary relationships were studied. Potato and tomato contained most of the homologous genes.

The chromosomal distribution of StAAAP genes in the genome was determined by extracting chromosomal data and mapping using Tbtools II. The distribution of the StAAAP gene on the chromosome is shown in Fig 3A. These 56 StAAAP genes were mapped unevenly to 12 chromosomes (Fig 3A). Chromosome 3 had most of the StAAAP genes (11), followed by chromosomes 2, 4, and 5 (7). Chromosome 8 had the fewest StAAAP genes, with only one gene. To identify the repetitive events in AAAP genes in potato, we analyzed the full-length sequences of 56 AAAP proteins using MCScanX. Six genes (28.57%) were arranged in tandem repeats and were divided into eight groups. Two pairs of tandem repeat genes were identified on chromosomes 2 and 4. Chromosomes 3, 5, 6, and 7 contained one pair each. This uneven distribution pattern was most likely influenced by chromosome length and tandem gene replication. The MCScanX function in TBtools demonstrated the collinearity of the potato AAAP genes on the chromosomes. There were ten pairs of collinear genes in the potato StAAAP gene family, and the results are shown in Fig 3B. In addition, interspecies collinearity maps of potato AAAP family genes with *A. thaliana Capsicum_annuum* (pepper), *Nicotiana attenuata* (tobacco), and *Solanum_lycopersicum* (tomato) were used to study the evolutionary relationship between species (Fig 3C). Potato and tomato contained the most homologous genes, with 38 pairs. Chili peppers contained 20 pairs of homologous genes, whereas potato and *A. thaliana* contained 17 pairs of homologous genes. Potato and tobacco contained the fewest pairs of homologous genes. The results showed a close genetic relationship between potato and tomato AAAP genes.

### 3.4. Prediction of cis-acting elements of StAAAP genes

**Fig 4:**
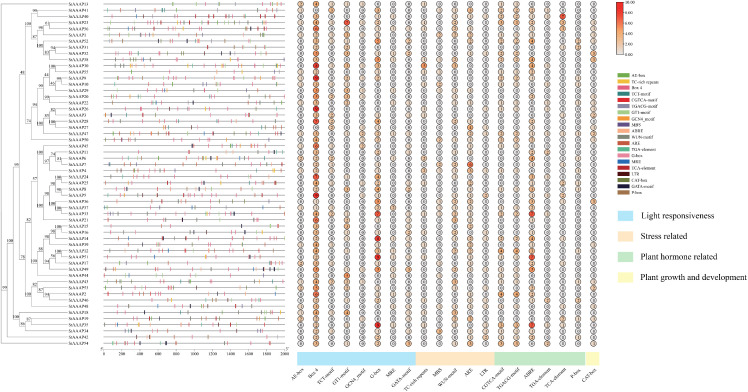
Original analysis of the cis-acting 2,000 bp promoter in the StAAAP genes. To further understand the potential functions of StAAAP, the 2,000 bp promoter sequence upstream of the StAAAP genome was analyzed and the cis-acting elements of StAAAP were detected. Different colors in the figure represent different cis-acting elements, including the AE-box, TC-rich repeats, Box 4, TCT-motif, and CGTCA-motif.

Cis-acting elements provide important clues for predicting gene functions. Transcription factors influence the expression levels of target genes by binding to cis-acting elements of target genes during specific biological processes. To further investigate the function of StAAAP genes, we used the PlantCARE database to predict the cis-acting elements in its putative promoter region of StAAAP genes. The promoter sequence 2,000 bp upstream of the StAAAP genome was analyzed. A total of 552 cis-acting elements were identified, and the results are shown in Fig 4. These elements are mainly related to hormones, plant growth, and plant development. ABRE is related to abscisic acid response, CGTCA-motif and TGACG-motif are related to jasmonic acid response, TGA-element is related to auxin response, AE-box and T-C rich repeats are related to light response, and ARE is related to anaerobic induction. MRE and LTR are related to the low-temperature response, whereas CAT-box and GCN4-motif are related to growth and development. In our study, cis-elements related to anaerobic induction were identified among the 40 AAAP genes. Cis-elements related to jasmonic acid were identified in 33 genes, whereas cis-elements related to abscisic acid were identified in 31 genes. In summary, the StAAAP gene family of potatoes plays an important role in the growth and development of potato as well as in the response to abiotic stress.

### 3.5. Transcriptomic analysis of StAAAP genes

**Fig 5:**
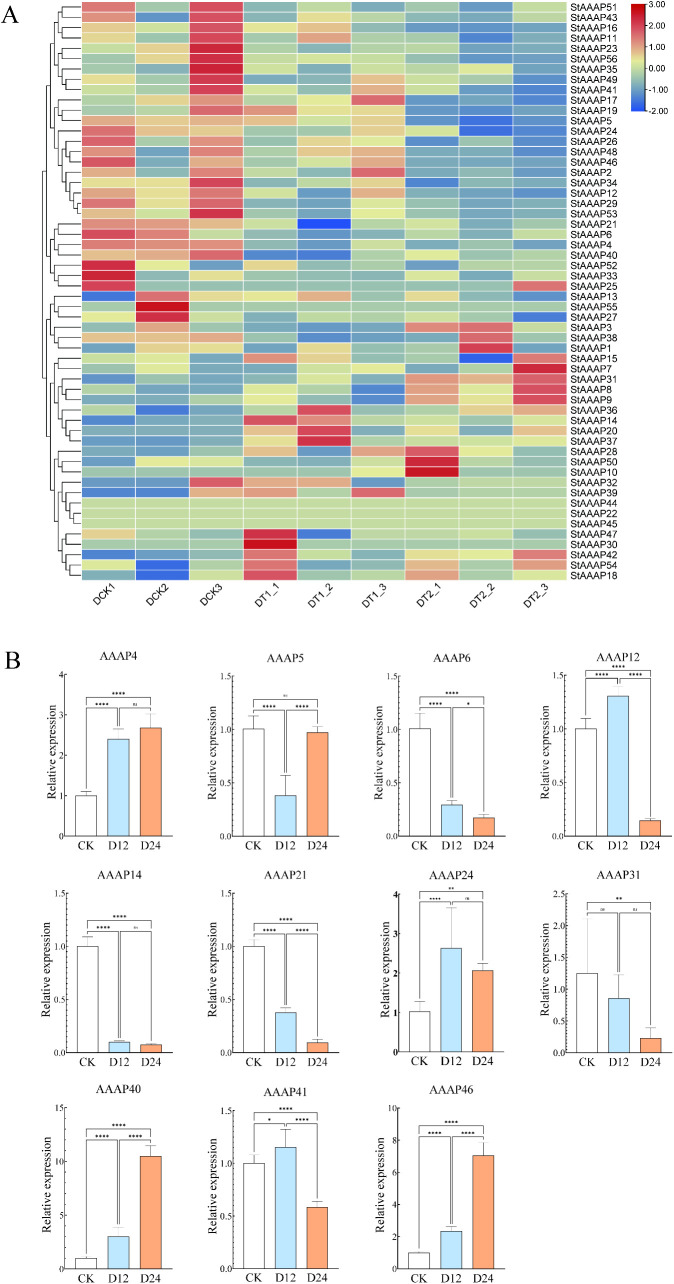
Verification of gene expression and qRT-PCR under drought stress conditions. A: AAAP gene expression map of the potato plants subjected to drought stress. B: The expression of 11 AAAP genes analyzed by qRT-PCR under simulated drought stress. Panel A shows the expression heat map of each gene under drought stress, with red indicating upregulated expression and blue indicating downregulated expression. Combined with qRT-PCR analysis, three treatments (control and 12 h drought stress, and 24 h drought stress) were used to analyze the expression levels of genes.

**Fig 6:**
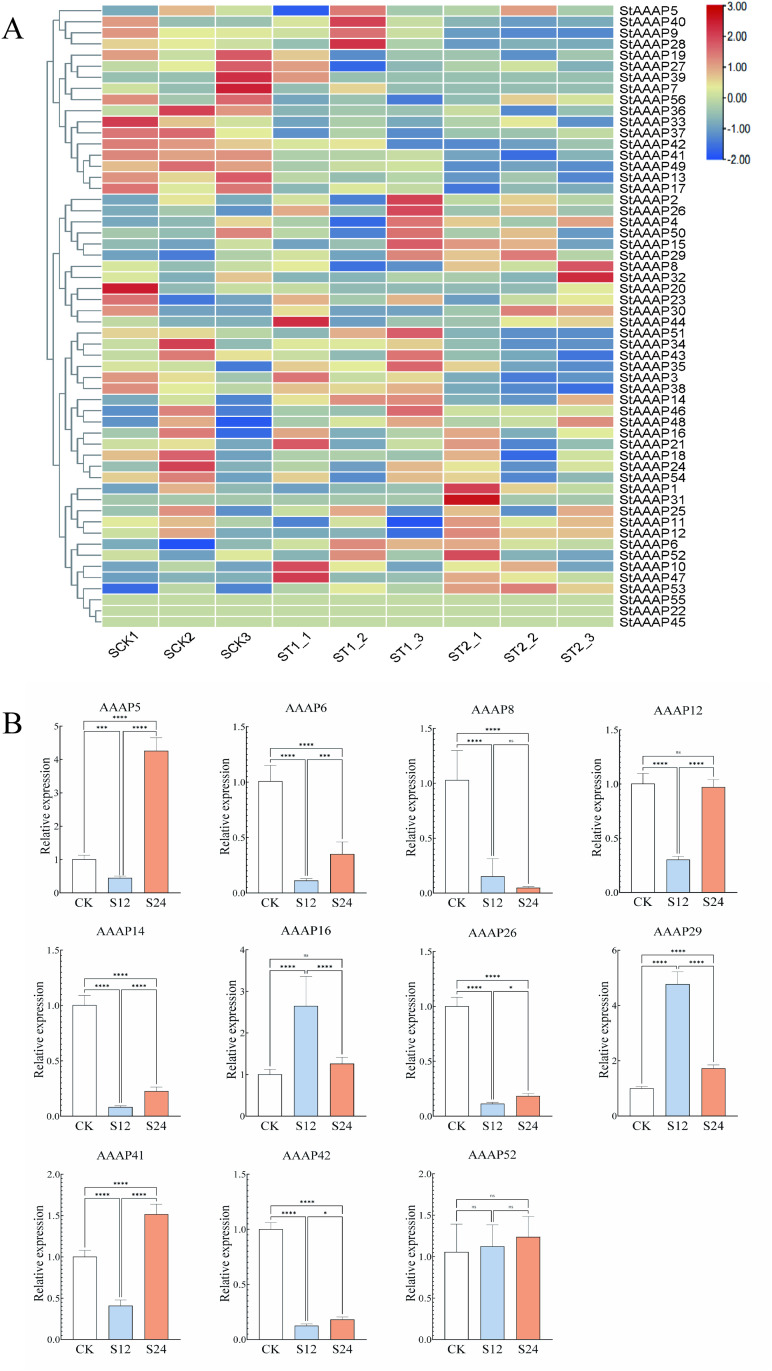
Gene expression and qRT-PCR verification under salt-stress conditions. A: AAAP gene expression map of potato under salt stress. B: The expression of 11 AAAP genes analyzed by qRT-PCR under simulated salt stress. Panel A shows the expression heat map of each gene under salt stress, with red indicating upregulated expression and blue indicating downregulated expression. Combined with qRT-PCR analysis, three treatments, namely, control, 12 h drought stress, and 24 h drought stress, were set up to analyze the expression levels of genes under salt stress.

To investigate whether StAAAP is involved in the response to abiotic stress in potato, we investigated the expression level of StAAAP in potato roots under drought and salt stress treatments, with the results shown in Figs 5A and 6A.The expression patterns of potato AAAP family members were analyzed using RNA-seq data downloaded from the NCBI database (entry numbers: PRJNA882516 and PRJNA646050). Compared with the control group, most potato AAAP genes, except for StAAAP45, StAAAP55, and StAAAP22, were induced in at least one treatment. Under drought stress, StAAAP family genes showed differential expression, with 23 genes upregulated and 29 genes downregulated (Fig 5A). It is speculated that the upregulated gene family members may be more sensitive to drought stress and respond in time to cope with the harm caused by drought stress. Under salt stress, the expression of 24 genes was upregulated and that of the remaining 29 genes was downregulated (Fig 6A). It is also speculated that the upregulated gene family members may be more sensitive to salt stress and respond in time to cope with the harm caused by salt stress. In summary, combined with the differential expression profile analysis of StAAAP genes under abiotic stress in potato transcriptome data, it is speculated that members of this gene family have important functions in potato stress-resistance regulatory networks, and many StAAAP genes may respond to abiotic stress.

### 3.6. qRT-PCR verification of StAAAP genes

To further verify the reliability of the StAAAP transcriptome data, we selected 11 genes for qRT-PCR under different stresses and analyzed their expression patterns. The results are shown in Figs 5B and 6B. Eleven genes were selected under drought stress: StAAAP4, StAAAP5, StAAAP6, StAAAP12, StAAAP14, StAAAP21, StAAAP24, StAAAP31, StAAAP40, StAAAP41, and StAAAP46. Under salt stress, 11 genes were also selected: StAAAP5, StAAAP6, StAAAP8, StAAAP12, StAAAP14, StAAAP16, StAAAP26, StAAAP29, StAAAP41, StAAAP42, and StAAAP52. The results showed that under drought stress, StAAAP genes were differentially expressed: StAAAP4, StAAAP24, StAAAP40, StAAAP41, and StAAAP46 were upregulated, whereas StAAA6, StAAA14, StAAA21, and StAAA31 were downregulated(Fig 5B). The expression trends of StAAAP5, StAAAP6, StAAAP12, and StAAAP21 were consistent with those of the transcriptome data, whereas the expression trends of the rest of the genes were different from those of the transcriptome data, which may be related to the different potato varieties. It is speculated that the expression of StAAAP genes differs among potato varieties. Under salt stress, the expression of StAAAP5, StAAAP16, StAAAP29, StAAAP41, and StAAAP52 was upregulated, whereas that of StAAAP6, StAAAP8, StAAAP14, StAAAP26, and StAAAP42 was downregulated(Fig 6B). The expression profiles obtained by qRT-PCR were consistent with those obtained by RNA-seq. In summary, StAAAP4, StAAAP24, StAAAP29, StAAAP40, and StAAAP46 genes may play key roles in potato abiotic stress responses, indicating high reliability of RNA-seq data.

## 4. Discussion

As an important gene family, AAAP plays an important role in all aspects of plant growth and development and is found in cayenne pepper [25], poplar [26], alfalfa [10], and bamboo [27]. Potatoes play an important role in food production and life worldwide. Therefore, to study the potential role of the AAAP gene family in stress response, we analyzed the potato AAAP gene family under different abiotic stress environments and verified its regulatory function under abiotic stress.

The doubling of gene families may be caused by self-replication of genes on the same chromosome, resulting in two or more closely spaced concatenations [28], or by structural changes in chromosomes during the evolution of species [29]. Gene replication plays an important role in genome evolution, expansion, and functional differentiation of species [30]. Studies have shown that 33.96% (18/53) of AAAP genes in pepper [25], 50% (29/58) in rice [13], and 30.43% (14/46) in Arabidopsis [11] are duplicates. In this study, 56 potato genes were distributed on 12 chromosomes; eight tandem repeat genes and 10 pairs of fragment repeat genes were identified on six chromosomes. Among potato AAAP genes, 58.93% (33/56) were repetitive genes, 16 (28.57%) were involved in tandem repetitions, and 17 (30.36%) were involved in fragment repetitions. These results indicate that gene tandem and fragment repeats exist between potato StAAAP genes and play an important role in the expansion of the potato StAAAP gene family. Fragment duplication is the main reason for the expansion of the AAAP gene family in potato, which is consistent with previous results in rice [13].

In this study, 552 cis-acting elements of the StAAAP conjectural promoter region were identified using the PlantCARE database, suggesting that StAAAP genes may play a complex and critical role in plant activities. Among them, cis-elements related to jasmonic acid and abscisic acid were present in 33 and 31 AAAP genes, respectively. Jasmonic acid and abscisic are important plant hormones involved in plant defense response, growth, development, stress response, and other processes [31]. The presence of the associated cis-elements indicates that these StAAAP genes may be involved in the response of plants to stress. In addition, the promoter region of StAAAP contains several elements related to anaerobic induction, light response elements, plant growth and development, and transcription factor-binding sites, suggesting that StAAAP gene family members may regulate plant adaptation to the external environment by responding to different environmental stimuli. Studies in millet [32] also showed that the AAAP gene family contains the corresponding stress-responsive cis-acting elements that regulate plant growth and development under abiotic stress. In summary, the potato AAAP gene family may be involved in hormone regulation as well as plant growth and development.

In this study, 56 potato AAAP genes were analyzed and a phylogenetic tree was constructed. Potatoes contain all eight subfamilies of the plant AAAP gene family, and the evolutionary branching pattern is consistent with the results for tobacco. Analysis of the StAAAP gene structure and conserved motifs showed that the gene structure and conserved motifs in the same subfamily were similar, indicating that genes in the same subfamily may have similar functions in potato physiological activities. However, there were differences among different subfamilies, indicating that genes in different subfamilies may have different functions in potato physiological activities. At the same time, subcellular localization results showed that StAAAP family members were mainly distributed in the plasma membrane, which was compatible with the long-distance amino acid transport by amino acid/auxin permease proteins. The presence of multiple collinear gene pairs between potato and *C. annuum*, *N. attenuata*, or *S. lycopersicum* suggest that these homologous genes may have similar functions in these species. In tobacco, NtAAP2−2 is involved in the transport of Glu, Gln, Asp, and Asn, and members of the AAP subfamily can transport positively charged and alkaline amino acids under neutral conditions [14]. It is predicted that members of the potato AAP subfamily have similar functions.

In plants, many stress-related genes produce a series of responses to cope with the adverse environments encountered during growth and development. AAAP genes are highly regulated by environmental signals and play active roles in abiotic stress responses in many plants [33,34]. Under abiotic stress, potato had at least 52 genes regulated in one of the treatments compared with the control. The proline transporter (ProT) has been shown to be located on the plasma membrane [11,35] and may be involved in the intercellular and long-distance transport of proline [11]. The localization of potato ProT subfamily to the plasma membrane in this paper is consistent with the conclusion. Studies have shown that HvProT and AtProT2 strongly respond to salt stress [36,37]. ClProT was found to play an important role in proline accumulation and alkali stress response, and ClProT2 can be used to genetically modify chrysanthemum stress resistance [35]. Studies have also shown that increased proline content can increase potato tolerance to adversity [38], and genes in the ProT and AAP subfamilies have been shown to respond to stress [25]. These results indicate that the potato AAAP gene family plays different roles in abiotic stress resistance.

The transcriptome results of this study showed that StAAAP14 and StAAAP31 were significantly expressed under drought stress, showing significant downregulation under simulated drought stress. The upregulated expression of StAAAP4, StAAAP24, StAAAP40, StAAAP41, and StAAAP46 under simulated drought stress was verified by RT-qPCR. It was speculated that under drought stress, the amount of water available for plants to absorb decreased, which led to the differential expression of StAAAP. StAAAP5, StAAAP16, StAAAP29, StAAAP41, and StAAAP52 were upregulated under salt stress, and the RT-qPCR results were consistent with the transcriptome expression trend. In conclusion, StAAAP genes are a key gene family involved in potato growth, development, and abiotic stress response. Under drought stress, the water content of the environment decreased significantly, affecting the water absorption of potatoes, and the expression of StAAAP family members changed significantly to adapt to the water shortage. Under salt stress, the concentration of ions in plants sharply increases. To adapt to the environment, the StAAAP gene family showed a trend of upregulated expression, which effectively alleviated the effect of salt stress on potatoes. These results indicate that the potato AAAP gene family plays different roles in abiotic stress resistance, which provides an important reference for further studies on its role in plant stress resistance mechanisms.

## 5. Conclusion

In this study, the AAAP gene family was comprehensively analyzed in potatoes. A total of 56 StAAAP gene family members were identified at the whole-genome level in potatoes and further divided into eight main groups. Tandem repeats and repeat fragments were observed between potato StAAAP genes, which played important roles in the expansion of the potato StAAAP gene family. Collinearity analysis and phylogenetic comparison of the StAAAP genes in several different plants provided valuable clues for understanding the evolutionary characteristics of the StAAAP gene family in potato. The StAAAP gene family plays an important role in the growth and development of potato and is widely involved in hormone regulation and plant growth and development. StAAAP genes are differentially expressed under different stress conditions to cope with the harmful effects of abiotic stress. The genes StAAAP4, StAAAP24, StAAAP29, StAAAP40, and StAAAP46 likely play key roles in the abiotic stress response of potato. These results provide a valuable resource for a better understanding of the biological roles of individual StAAAP genes in potatoes.

## Supporting information

S1 Table Primer information. It includes all the primer information for drought stress and salt stress, as well as the internal reference *StEF-1α*.
(XLSX)
